# High Exposure to *Toxoplasma gondii* and *Neospora* Spp. in Donkeys in Israel: Serological Survey and Case Reports

**DOI:** 10.3390/ani10101921

**Published:** 2020-10-19

**Authors:** Sharon Tirosh-Levy, Amir Steinman, Avital Minderigiu, Ori Arieli, Igor Savitski, Ludmila Fleiderovitz, Nir Edery, Gili Schvartz, Monica Leszkowicz Mazuz

**Affiliations:** 1 Koret School of Veterinary Medicine, The Robert H. Smith Faculty of Agriculture, Food and Environment, The Hebrew University of Jerusalem, Rehovot 7610001, Israel; sharontirosh@gmail.com (S.T.-L.); amirst@savion.huji.ac.il (A.S.); avitalmind@gmail.com (A.M.); oriarieli6@gmail.com (O.A.); giliun@gmail.com (G.S.); 2 Division of Parasitology, Kimron Veterinary Institute, Bet Dagan 50250, Israel; igors@moag.gov.il (I.S.); ludaf@moag.gov.il (L.F.); 3 Division of Pathology, Kimron Veterinary Institute, Bet Dagan 50250, Israel; nire@moag.gov.il; 4 Division of Virology, Kimron Veterinary Institute, Bet Dagan 50250, Israel

**Keywords:** *Toxoplasma gondii*, *Neospora*, donkey, IFAT, serology

## Abstract

**Simple Summary:**

*Toxoplasma gondii* and *Neospora* spp. are major pathogenic parasites of animals worldwide, with the first also affecting humans. These parasites have two-host life cycles, with the cat and the dog being the definitive hosts of *T. gondii* and *N. caninum*, respectively. Both parasites can infect various animal species, as intermediate hosts, in which they form tissue cysts and may cause abortions and neurological disease. Both parasites have been reported in wild and domestic animals in Israel. This study aimed to evaluate the serologic exposure of donkeys to these parasites. A total of 98 donkeys were examined. Half of them (*n* = 49) were from animal shelters in Israel, and the rest (*n* = 49) were working donkeys from the Palestinian Authority. Anti-*T. gondii* antibodies and anti-*Neospora* spp. antibodies were found in 94% and in 70% of the donkeys, respectively. In addition, two cases of donkeys presenting *N. caninum* tissue cysts, which were detected during post-mortem examination, were described. This is the first report of the exposure of donkeys to *Toxoplasma gondii* and *Neospora* spp. in the area. The exposure of donkeys to both parasites was considerably higher than the exposure of other species in the area and may be the result of poor husbandry conditions and higher exposure to infection. These results indicate that donkeys may have an important role in the maintenance and transmission of these parasites.

**Abstract:**

*Toxoplasma gondii* and *Neospora* spp. are closely related cyst-forming coccidian parasites, which infect various animal species and have considerable zoonotic and economic implications, respectively. Both parasites are endemic in Israel and have been reported to infect wild and domestic animals. This study was conceived to evaluate the serologic exposure of donkeys to these parasites. Serum samples were collected from 98 donkeys. Half of them (*n* = 49) were from animal shelters in Israel, and the rest (*n* = 49) were working donkeys from the Palestinian Authority. The donkeys were screened for the presence of anti-*Toxoplasma* and anti-*Neospora* antibodies by immunofluorescence antibody tests (IFATs). The seroprevalence of *T. gondii* and *Neospora* spp. was 94% and 70%, respectively, and 69% of the donkeys were exposed to both parasites. In addition, *N. caninum* tissue cysts were documented in two donkeys during post-mortem examination. This is the first report of the exposure of donkeys to *T. gondii* and *Neospora* spp. in the area. The high prevalence found in this study suggests that donkeys may have a role in the maintenance of these parasites in the area, thus serving as a source of infection for the definitive hosts.

## 1. Introduction

Cyst-forming coccidian parasites, mainly *Toxoplasma gondii* and *Neospora* spp., are major pathogens of animals with worldwide distribution. These closely related apicomplexan intracellular parasites have heteroxenous life cycles, with felids being the definitive hosts of *T. gondii* and canids of *N. caninum*. Both parasites infect various mammalian species as intermediate hosts in which they form tissue cysts [1]. 

Toxoplasmosis may cause abortions, fetal damage or neurologic disease in a wide range of animals and humans. It is a prominent cause of abortion in sheep and a common zoonosis [1,2,3,4]. The clinical significance of *T. gondii* infection in horses is unclear. Nevertheless, horses may be exposed to or infected by the *T. gondii* parasite with no apparent clinical signs, and may pose a zoonotic risk through the consumption of infective horse or donkey meat [5,6,7]. In Israel, the seroprevalence of *T. gondii* was evaluated as 20–60% in humans [8], 25% in sheep [9], 36% in dogs [10], 17% in cats [11], 43% in crows, 40% in Griffon vultures [12] and 2.5% in horses [13].

Neosporosis causes abortion and neurologic disease in various animals. *Neospora caninum* is a major cause of abortion and economic loss in the cattle industry, and has been reported in horses [1,14]. *Neospora hughesi* is known to affect only equids and was isolated from cases of neurological disease in horses [14,15]. These two species are indistinguishable when using standard serological methods [16]. Horses may be infected with both parasites, and since most surveys were based on serology, the reported prevalence in horses and donkeys was of *Neospora* spp. In Israel, the seroprevalence of *Neospora* spp. was evaluated as 51% in cattle [17], 1.3–67% in wild animals [18] and 12% in horses [19]. The seroprevalence in aborting mares and cows was significantly higher than in the general population [17,19]. 

Domestic donkeys (*Equus africanus acinus*) in Israel are used as draught animals, riding animals, as pets and in petting zoos, and therefore are in close contact with humans. In other countries, donkeys are also used for their meat as food, and certain donkey-derived items are also important for traditional Chinese medicinal purposes [20]. Since donkeys are closely related to horses, they may be affected by similar pathogens. Exposure to both parasites has been reported in donkeys worldwide [5,6,21,22,23,24,25,26,27]. However, little is known about the role of donkeys in the epidemiology of these parasites. In Israel, data concerning the exposure of donkeys to infectious diseases are scarce. The aim of this study was to assess whether donkeys in Israel are exposed to *T. gondii* and *Neospora* spp. protozoan parasites, and to report two neosporosis cases of affected donkeys.

## 2. Materials and Methods

### 2.1. Sample Collection for Serological Survey

The sample size was calculated according to the prevalence of *Neospora* spp. in horses in Israel (12%) [19] using WinPepi 11.43^®^. A sample size of 77 donkeys met the criteria for an expected prevalence under 30%, with a relative error of ≤20% and 95% confidence level. 

Since no data are available for estimating the distribution of donkeys in Israel, it was unfeasible to design a sample that would reliably represent the donkey population in the area. Therefore, donkeys were sampled at two donkey shelters in Israel that receive donkeys from different locations (49 donkeys), and at three locations in the Palestinian Authority (PA) to which working animals were brought to receive veterinary care given through a humanitarian association (49 donkeys). 

Blood collections were performed with owners’ consent, and the study was approved by the Internal Research Review Committee of the Koret School of Veterinary Medicine—Veterinary Teaching Hospital (KSVM-VTH/23_2014). Blood was collected from the jugular vein of each animal into a sterile vacuum tube without anticoagulant. Sera were obtained from the clotted blood samples by centrifugation (4000× *g* for 10 min) and stored at −20 °C until processing. During sample collection, data for each donkey were recorded including the farm’s location, sex, age, and origin of the donkey, when available. At the time of sampling, all donkeys were apparently healthy according to both the owners and the veterinarians who collected the samples.

### 2.2. Serological Screening Using Immunofluorescence Antibody Test (IFAT)

Serological screening for the presence of anti-*T. gondii* antibodies was conducted on 1:2 serial dilutions of the sera, starting at 1:64 as a cut-off value for screening [28,29], up to a final dilution of 1:16,384.

Serological screening for the presence of anti-*Neospora* spp. antibodies was conducted on 1:2 serial dilutions of the sera, starting at 1:50 as a cut-off value for screening [19,25,29], up to a final dilution of 1:3200.

In-house antigens were prepared as previously described [30]. Briefly, free tachyzoites were obtained from an infected Vero cell culture, separated by centrifugation, diluted in phosphate buffered saline (PBS), dropped onto slides and stored at −80 °C until use. The slides were thawed at 37 °C for 30 min and air dried. The sera were diluted in PBS with 1% bovine serum albumin (BSA). A volume of 35 µL of serum was added to each antigen drop well and incubated in a humid chamber at 37 °C for 30 min. The slides were washed for 10 min in carbonate buffer (pH 9, diluted 1:4 in distilled water) before the application of 35 µL of anti-horse fluorescein isothiocyanate (FITC) secondary antibody (Sigma-Aldrich, St. Louis, MO, USA) diluted 1:80 with PBS–BSA, and incubation at 37 °C for 30 min in a humid chamber. The slides were later washed for 10 min in carbonate buffer, dried, mounted with glycerol/carbonate buffer (50%) and examined under a fluorescence microscope. Positive and negative control serum samples from positive horses were added to each run.

### 2.3. Statistical Analysis of Serology Results

Risk factors associated with exposure to either parasite or both parasites were assessed separately and included the farm, the geographical area (Israel versus the Palestinian Authority), the donkey’s sex and age. Association with nominal independent variables was assessed by using the χ^2^ test or Fisher’s exact test, as appropriate, and odds ratios were calculated. Association with quantitative parameters was assessed using *t*-tests. Association between potential risk factors and antibody titer was assessed using ANOVA. Statistical significance was set at *p* < 0.05. The analysis was performed using the SPSS 22.0^®^ and Win Pepi 11.43^®^ statistical software.

### 2.4. Sample Collection, Histopathology and Polymerase Chain Reaction (PCR) of Clinical Samples

Skeletal muscle tissue from the triceps brachi muscle was collected from two donkeys (Donkeys 1 and 2) and was sent to the Kimron Veterinary Institute pathology department for post-mortem examination. Brain tissue was collected from one of these donkeys (Donkey 1), which was reported to have neurological signs prior to euthanasia, and stored at −20 °C until processing. Muscle tissue samples were transferred into 4% formaldehyde solution before the preparation of histological slides with hematoxylin and eosin (H&E) staining.

DNA was extracted from the paraffin-embedded muscle tissue dissolved in xylene and washed with PBS, using the DNeasy blood and tissue kit (QIAGEN, Hilden, Germany) according to the manufacturer’s instructions. The presence of coccidian parasites was confirmed using PCR targeting the small-subunit rRNA (COC1 F: AAGTATAAGCTTTTATACGGCT, COC2 R: CACTGCCACGGTAGTCCAATAC), and the classification of the species was achieved by sequence- and species-specific PCRs targeting *Besnoitia besnoiti*, *Hammonidia*, *N. caninum, Sarcocystis* spp., *T. gondii* and *Trypanosoma* spp. (the primers and targets are specified in Table 1).

RNA was extracted from brain tissue, and the presence of West Nile virus (WNV) RNA was tested for and confirmed by real-time reverse transcription PCR (qRT-PCR) and further amplified and sequenced, as previously described [31].

All positive PCR products were sent for sequencing by HyLabs (Rehovot, Israel). Sequences were evaluated using the Chromas software (Technelysium Pty Ltd., Tewantin, QLD, Australia, version 2.6) and assembled using the MEGA7 software (http://www.megasoftware.net, version 7.0.18). Consensus sequences were created for each gene from both donkeys and were submitted to GenBank.

## 3. Results

### 3.1. Serologic Exposure to T. gondii and Neospora Spp. in Donkeys

The study population comprised donkeys from Israel (*n* = 49) and the Palestinian Authority (*n* = 49). Sixty of the donkeys were males (61%), and 38 were females (39%). Age was available for 70 of the donkeys and ranged between four months and 25 years (mean = 7.6 years, median = 7.0 years, standard deviation = 5.1 years). All donkeys were apparently healthy during blood collection.

**Figure 1:**
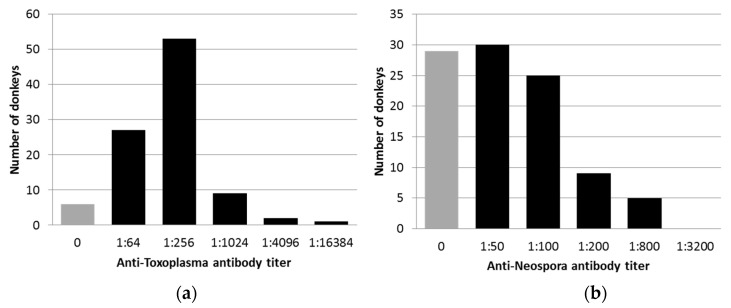
The distribution of anti-*T. gondii* (**a**) and anti-*Neospora* spp. (**b**) antibody titers in 98 donkeys, as detected by immunofluorescence antibody tests (IFATs).

Anti-*T. gondii* antibodies were detected in 92 of 98 donkeys (94%). The antibody titers ranged between 1:64 and 1:16,384 (Figure 1a), while high titers (≥1:256) were found in 66% of the animals. No significant risk factors for exposure were identified.

Anti-*Neospora* spp. antibodies were detected in 69 of 98 donkeys (70%). The antibody titers ranged between 1:50 and 1:800 (Figure 1b), while high titers (≥1:200) were found in 14% of the animals. No significant risk factors for exposure were identified. High antibody titers (≥1:200) were associated with one farm in Israel. In this farm, 10 out of 25 donkeys had high antibody titers, representing 71.4% (10 of 14) of the donkeys with high antibody titers (*p* > 0.001, odds ratio (OR) = 11.5, 95% confidence interval (CI) = 2.76–55.29).

The majority of donkeys (68/98, 69.4%) were exposed to both parasites. An additional 24 donkeys (24.5%) were exposed only to *Toxoplasma gondii*, one donkey (1%) was exposed only to *Neospora* spp., and five donkeys (5.1%) were not exposed to any of these parasites. No significant risk factors for co-exposure were identified.

### 3.2. Clinical Cases of Neosporosis in Donkeys

**Figure 2:**
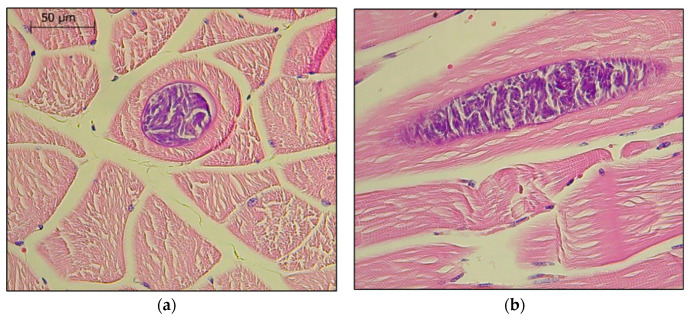
*Neospora caninum* tissue cysts in the skeletal muscles of the front limb of Donkey 1: (**a**) transverse section; (**b**) longitudinal section. Giemsa, ×1000.

Parasitic tissue cysts were identified in the skeletal muscles of two donkeys sent for post-mortem examination (Figure 2). Both donkeys were sent from the same animal shelter. The first donkey (Donkey 1) was over 30 years old and presented with neurological signs and severe weakness before euthanasia. The second donkey (Donkey 2) was over 20 years old, and his left thoracic limb had been amputated several years prior to his death from unrelated causes. After his demise, the right thoracic limb was sent for evaluation for any potential effect of the amputation on the contralateral limb. The donkey that had presented with neurological signs (Donkey 1) was diagnosed as being infected with WNV after viral RNA was isolated and sequenced from its brain tissue (MT828577). In both donkeys, multifocal tissue cysts containing parasites were observed (Figure 2). *Neospora caninum* DNA was identified in the skeletal muscle tissue of both donkeys and was confirmed by the sequencing of two different species-specific target genes (NC5 (MT831977) and ITS1 (MT826198), over 97% homology with species-specific sequences in GenBank). Specific PCRs targeting *Besnoitia* spp., *Hammonidia*, *T. gondii*, *Neospora* spp., *Sarcocystis* spp., *T. gondii* and *Trypanosoma* spp. were negative, and the presence of these related cyst-forming parasites was ruled out.

## 4. Discussion

The seroprevalence of both *T. gondii* and *N. caninum* in donkeys in Israel is high, and higher than the recorded prevalence in any other mammalian species in the area, including horses [13,18]. Donkeys in Israel are sometimes kept as burden animals, being more prevalent in Arab and Bedouin villages and often receiving little veterinary care, in comparison to horses. Half of the donkeys in this survey were sampled in animal shelters that receive neglected donkeys from various locations, while the other half were sampled in Arab villages in the Palestinian authority, by a veterinarian giving free veterinary care through a humanitarian organization. The high exposure to both parasites may be the result of the poor sanitation associated with low-income populations, which may increase the chance of exposure to oocysts in water sources or the environment. The association between low income and higher exposure to *T. gondii* has been previously described in humans and in horses [7,37,38]. In addition, in poor sanitary conditions, stray dogs and cats may have access to and feed on donkey carcasses, thus enhancing the maintenance and transmission of Neosporosis and Toxoplasmosis in these areas.

The seroprevalence of *T. gondii* in horses varies between countries and ranges between 1.2% in Sweden [39] and 71.2% in Iran [40]. The differences in prevalence may be associated with housing, stable hygiene and feeding practices [7,41]. The prevalence of *T. gondii* was higher in donkeys (72.7%) than in horses (27.7%) in a recent study from Brazil [42], as well as in other studies from Spain [23] and Pakistan [43]. However, since there are fewer studies evaluating both horses and donkeys in similar cohorts, it is difficult to determine whether these differences reflect a higher susceptibility of donkeys, or merely reflect differences in sanitation or management practices between these species. Since horses are considered to be naturally resistant to *T. gondii* infection [42], it is possible that they develop lower antibody titers that are not detected by serological tests.

In Israel, *T. gondii* seroprevalence was significantly higher in the Arab population (60.4%) than in Bedouins (27.5%) or Jews (19.9%) [8], and similar to the reported prevalence in Lebanon (62.2%) [44]. The seroprevalence was age-dependent and reached 96% in Arabs over 60 years of age [8]. The differences within ethnic groups in Israel may be attributable to a combination of economic status, exposure to animals and climate [8]. Since donkeys in Israel are more abundant in Arab settlements, the high seroprevalence may reflect similar conditions for exposure.

Although the consumption of donkey or horse meat is not common in this area, infection from contaminated meat is still a potential source for zoonotic transmission. Viable *T. gondii* parasites have been isolated from horse and donkey meat intended for human consumption worldwide [5,6,45,46,47], with a possible link to human disease [46].

In a recent study [48], an association between *T. gondii* seropositivity and the prevalence of impaired cognitive function was demonstrated in humans. Researchers theorize that behavior manipulation increases the parasite’s likelihood of transmission by manipulating the host to engage in risky behaviors so that the host is likely to be preyed upon, particularly by a feline [48]. This was demonstrated in rats [49]. In this study, behavioral changes were not observed in any of the animals. To the best of our knowledge, behavioral changes have never been reported in equids in regard to *T. gondii* infection.

Neosporosis is not considered zoonotic, and its main impact is economic, mostly due to its effect on the reproduction of cattle and small ruminants, which are considered as the main intermediate hosts [14,50]. Therefore, fewer data are available regarding its prevalence in equines. In Israel, the prevalence in horses (12%) [19] was considerably lower than our findings in donkeys (70%). In neighboring Jordan, the reported seroprevalence in horses was 32% [51], and that in small ruminants was 63% [52]. Both dogs and donkeys often accompany small ruminant flocks in the Middle East, which may increase the chance of infection of both the primary and secondary hosts. In 1998, a new species of *Neospora*, *N. hughesi*, was identified in a horse from California [15]. Since then, *N. hughesi* has been reported only in horses and mostly from North America; however, since the two *Neospora* species are indistinguishable serologically, the global distribution of *N. hughesi* is unclear [14]. In the two cases of tissue cysts described here, parasites were classified as *N. caninum* based on two loci. In addition, *N. caninum* was identified in several cases of *Neospora*-induced abortion in mares in Israel (Mazuz et al., unpublished data), while *N. hughesi* has never been described in the area.

In horses, both toxoplasmosis and neosporosis had been associated with neurological disease, while neosporosis had also been described as a cause of abortions and neonatal disease [53,54]. However, reports of equine clinical cases are rare, and the majority of seropositive horses are asymptomatic. The clinical significance of high exposure to these parasites in donkeys is unclear. All donkeys in this study were apparently healthy, and to the best of our knowledge, clinical toxoplasmosis or neosporosis in donkeys has never been reported in Israel or elsewhere. Although one of the clinical cases in this report (Donkey 1) exhibited marked neurological signs, the cause of the neurological disease was determined as WNV, which is the most common cause of neurological disease in horses in the area [55]. Nevertheless, the identification of *N. caninum* tissue cysts in muscle specimens from two donkeys suggests that donkeys may be susceptible to clinical infection.

## 5. Conclusions

This is the first epidemiological survey investigating the exposure of donkeys to *T. gondii* and *N. caninum* in Israel. The high seroprevalence of both parasites in donkeys may reflect a high susceptibility of this species or high exposure due to poor husbandry conditions. The pathological findings of *N. caninum* tissue cysts in two donkeys suggest that donkeys may also be clinically infected and highlight the potential of donkeys to be a source of infection to other animal species. The higher exposure of donkeys in relation to other animal species in these areas suggests that donkeys could be used as sentinels to monitor exposure to these important, and potentially zoonotic, parasites.

## Figures and Tables

**Table 1:** The PCR primers used to identify the coccidian species of the tissue cysts found in donkeys’ muscle tissue.

Organism	Primer	Sequence	Amplicon Size (bp)	Target Gene	Reference
*Besnoitia* spp.	ITS1-F	TGACATTTAATAACAATCAACCCTT	250	ITS	[32]
	ITS1-R	GGTTTGTATTAACCAATCCGTGA			
	Bes-F	ATTGGGACCGTTTTGTGG		ITS	
	Bes-R	CCTCTCGAGGCTACAAGTCG			
	Bes-F2	CCTCCTCACTCTGCTATCACG	750	(nested)	
	Bes-R2	TTCCACTGGTAACGCCTCT			
*Sarcocyst* spp.	71-F	CGGATCGCATTATGACCTTT		18S rRNA	
	894-R	GGTGCAGGAGAAGTCAAGGA			
	317-F	ATTGGAATGATGGGAATCCA	300	(nested)	
	548-R	TGCCACCAACACAATGAAGT			
*Toxoplasma gondii*	Tox4	CGCTGCAGGGAGGAAGACGAAAG	500	Non-coding	[33]
	Tox5	CGCTGCAGACACAGTGCATCTGG			
*Hammondia* spp.	JS4	CGAAATGGGAAGTTTTGTGAAAC	270	ITS	[34]
	JS5	CAGCAGCTACATACGTAGA			
*Neospora* spp.	476-F	CTGCTGACGTGTCGTTGT		NC5	[35]
	1014-R	CATCTACCAGGCCGCTCTTC			
	631-F	GCGTCAGGGTGAGGACAGTG	279	(nested)	
	910-R	CTCTCCGTTCGCCAGCAGTG			
	ITS1D-F	TACCGATTGAGTGTTCCGGTG		ITS	[36]
	ITS1D-R	GCAATTCACATTGCGTTTCGC			
	ITS1Di-F	CGTAACAAGGTTTCCGTAGG	480	(nested)	
	ITS1Di-R	TTCATCGTTGCGCGAGCCAAG			
*Trypanosoma* spp.	ITS1	GATTACGTCCCTGCCATTTG		ITS	
	ITS2	TTGTTCGCTATCGGTCTTCC			
	ITS3	GGAAGCAAAAGTCGTAACAAGG	1200	(nested)	
	ITS4	TGTTTTCTTTTCCTCCGCTG			
